# Renal Sympathetic Hyperactivity in Diabetes Is Modulated by 5-HT_1D_ Receptor Activation via NO Pathway

**DOI:** 10.3390/ijms24021378

**Published:** 2023-01-10

**Authors:** Juan Francisco Fernández-González, José Ángel García-Pedraza, José Luis Ordóñez, Anaïs Clara Terol-Úbeda, María Luisa Martín, Asunción Morán, Mónica García-Domingo

**Affiliations:** 1 Laboratorio de Farmacología, Departamento de Fisiología y Farmacología, Facultad de Farmacia, Universidad de Salamanca, 37007 Salamanca, Spain; 2 Instituto de Investigación Biomédica de Salamanca (IBSAL), Paseo San Vicente 58-182, 37007 Salamanca, Spain

**Keywords:** 5-HT_1D_ receptor, diabetes, diabetic complications, renal vasculature, sympathetic neurotransmission, vasopressor responses

## Abstract

Renal vasculature, which is highly innervated by sympathetic fibers, contributes to cardiovascular homeostasis. This renal sympathetic outflow is inhibited by 5-HT in normoglycaemic rats. Considering that diabetes induces cardiovascular complications, we aimed to determine whether diabetic state modifies noradrenergic input at renal level and its serotonergic modulation in rats. Alloxan diabetic rats were anaesthetized (pentobarbital; 60 mg/kg i.p.) and prepared for in situ autoperfusion of the left kidney to continuously measure systemic blood pressure (SBP), heart rate (HR), and renal perfusion pressure (RPP). Electrical stimulation of renal sympathetic outflow induces frequency-dependent increases (Δ) in RPP (23.9 ± 2.1, 59.5 ± 1.9, and 80.5 ± 3.5 mm Hg at 2, 4, and 6 Hz, respectively), which were higher than in normoglycaemic rats, without modifying HR or SBP. Intraarterial bolus of 5-HT and 5-CT (5-HT_1/5/7_ agonist) reduced electrically induced ΔRPP. Only L-694,247 (5-HT_1D_ agonist) reproduced 5-CT inhibition on sympathetic-induced vasoconstrictions, whereas it did not modify exogenous noradrenaline-induced ΔRPP. 5-CT inhibition was exclusively abolished by i.v. bolus of LY310762 (5-HT_1D_ antagonist). An inhibitor of guanylyl cyclase, ODQ (i.v.), completely reversed the L-694,247 inhibitory effect. In conclusion, diabetes induces an enhancement in sympathetic-induced vasopressor responses at the renal level. Prejunctional 5-HT_1D_ receptors, via the nitric oxide pathway, inhibit noradrenergic-induced vasoconstrictions in diabetic rats.

## 1. Introduction

In the 21st century, human well-being is threatened not only by emerging illnesses but also by chronic diseases, which entail a worldwide social, economic, and health problem. Diabetes, which is rapidly increasing all over the world, is one of the most feared ones, not only for the presence of hyperglycaemia as a result of insulin resistance or secretion failure [1], but also for the development of complications that lead to dysfunction and collapse of different organs and systems, such as kidney, blood vessels, or nerves [2,3,4]. Within this framework, diabetic nephropathy has become one of the most widespread chronic microvascular diabetic complications, which is characterized by progressive renal function decline [5]. Although the kidney is arguably the most important target of microvascular alterations in diabetes, the development of autonomic diabetic neuropathy in patients may contribute to this damage and originate dysregulation of cardiovascular homeostasis [6,7].

In this sense, noradrenergic innervation plays a crucial role both in renal, and in the whole cardiovascular system, regulation. At the renal level, the sympathetic nervous system is the main actor in blood pressure regulation, since it is involved in tubular sodium reabsorption, renin release, and renal vascular resistance. It is widely described that impairment of sympathetic activity is related to increased vasopressor responses, endothelial alterations, and augmented oxidative stress that come up with vascular damage [8,9]. Moreover, the increased sympathetic outflow may contribute to thrombogenesis as it disturbs platelet activation and aggregation [10]. All these interrelated dysfunctions have been described as diabetic complications [11,12,13].

The serotonergic system plays a pivotal function in cardiovascular homeostasis, both by directly regulating blood vessel contraction and dilation in different vascular beds such as mesentery, hindquarters, and kidneys [14,15,16], or by indirectly modulating the vegetative nervous system at the vascular and cardiac levels [17,18,19,20]. In this sense, 5-HT causes rat vascular sympatho-inhibition mainly by activation of 5-HT_1D_ receptors [18,19,21]. However, experimental diabetes, as well as pharmacological 5-HT modulation by the antagonism of 5-HT_2_ receptor, modify the serotonergic effect on the sympathetic discharge of rat vasculature [22,23,24,25], maintaining the main vascular sympatholytic effect of 5-HT. Furthermore, the inhibition of 5-HT reuptake during diabetes crucially modifies serotonergic regulation of the noradrenergic system, showing both sympatholytic and sympatho-excitatory action [26]. Serotonin is also able to play a neuroinhibitor role of sympathetic drive in different vascular beds (mesentery, renal); specifically, at renal level, our research group has shown that prejunctional 5-HT_1D_ receptor activation inhibits renal vasopressor noradrenergic outflow, involving nitric oxide (NO) production [18].

Considering that (a) diabetes is linked to long-term micro- and macrovascular problems that impair large blood vessels and the kidney (diabetic nephropathy), (b) the kidney is richly innervated by sympathetic fibers which are dysregulated during diabetes (diabetic neuropathy), (c) 5-HT_1D_ receptor activation, via the NO pathway, modulates noradrenergic input at renal level in normoglycaemic state, and that (d) presently, there is an active search for new therapeutical approaches to minimize renal damage in diabetes, it becomes urgent to explore the serotonergic system’s role in these complications to find new possible pharmacological targets. Thus, our work intended to determine whether the serotonergic system modulates noradrenergic input in rat renal vascular beds during diabetes, establishing the receptor type/subtype(s), nature, and possible endogenous mediators involved.

## 2. Results

### 2.1. Systemic Haemodynamic Parameters in Diabetic Rats

The administration of alloxan induced a hyperglycaemic state after two days; glycaemia increased from 112 ± 3 mg/dL (day 0) to 548 ± 7 mg/dL (day 2) (*p* < 0.05 vs. day 0). The hyperglycaemic state was maintained until day 28: 554 ± 6 mg/dL (*p* < 0.05 vs. day 0). In relation to body weight, there was significant decrease at day 2: 288 ± 2 g vs. 305 ± 1 g at day 0 (*p* < 0.05 vs. day 0) that was restored at day 28 (304 ± 3 g; no statistical differences vs. day 0).

After anaesthesia and surgical procedure, basal systemic blood pressure (SBP), renal perfusion pressure (RPP), and heart rate (HR) were 97 ± 3 mm Hg, 113 ± 2 mm Hg, and 308 ± 6 beats/min, respectively. These values were not significantly modified after i.v. administration of saline, other vehicles (ethanol (EtOH) 5%, HCl 0.01 M, or PEN, consisting in a mixture of polyethylene glicol 33%, EtOH 33%, 0.2 M NaOH 34%), serotonergic receptor type/subtype antagonists, glibenclamide, ODQ or indomethacin. Intraarterial administration of vehicles (saline, EtOH 5% or HCl 0.01 M; 10 μL) or serotonergic receptor type/subtype agonists did not modify per se any of the basal haemodynamic parameters. Only 5-HT at doses of 0.1 µg/kg and 0.4 µg/kg or α-methyl-5-HT (0.4 µg/kg) provoked significant increases in RPP (up to 142 ± 8.9, 175 ± 12.0 and 158 ± 17.2 mm Hg, respectively) that came back to basal values within 2 min.

### 2.2. Effects of Saline or 5-HT on the Electrically Induced ΔRPP in Diabetic Rats

The increases in RPP (ΔRPP) induced by electrical stimulation were frequency-dependent (23.9 ± 2.1, 59.5 ± 1.9 and 80.5 ± 3.5 mm Hg at 2, 4 and 6 Hz, respectively; stimulation response (S-R) curve E0). These vasopressor responses were due to selective stimulation since no effects were observed in HR or SBP. These values were not altered after i.a. administration of saline (Table 1), EtOH 5% or HCl 0.01 M.

5-HT at the doses of 0.0125, 0.1 and 0.4 µg/kg, administered intraarterially, significantly inhibited the ΔRPP in a dose- and frequency-dependent way (Table 1).

### 2.3. Effect of Serotonergic Receptor Type Agonists on the Increases in RPP Induced by Electrical Stimulation of Renal Periarterial Nerves in Diabetic Rats

**Figure 1:**
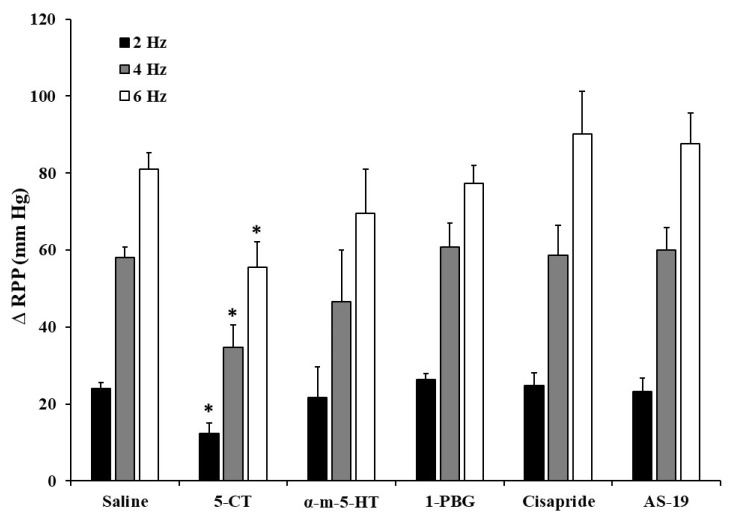
Effect of i.a. administration of serotonergic receptor agonists on the electrically induced increases in renal perfusion pressure in diabetic rats. Effect of i.a. bolus of saline (10 µL) or the serotonergic receptor agonists 5-CT (5-HT_1/5/7_), α-methyl-5-HT (5-HT_2_), 1-PBG (5-HT_3_), cisapride (5-HT_4_), or AS-19 (5-HT_7_) (0.4 µg/kg each agonist) on the vasopressor responses elicited by electrical stimulation of renal sympathetic nerves in diabetic rats. * *p* < 0.05 vs. saline. α-m-5-HT, α-methyl-5-HT; 1-PBG, 1-phenylbiguanide.

**Figure 2:**
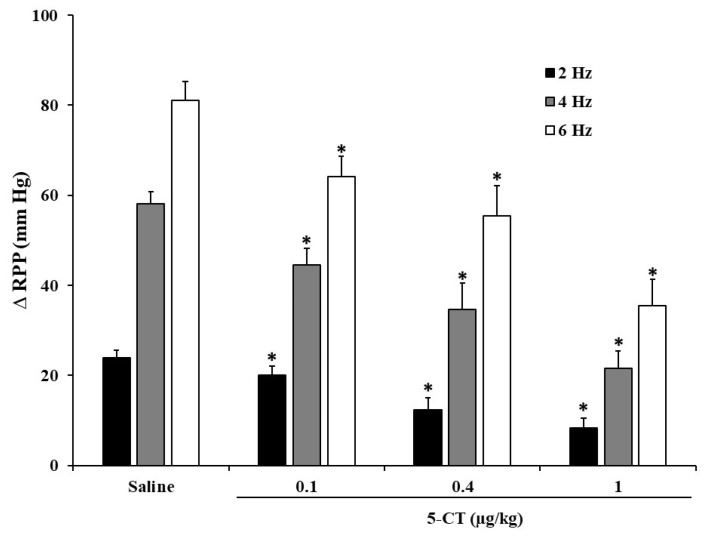
Effect of i.a. bolus administration of increasing doses of 5-CT on the vasopressor responses elicited by electrical stimulation of renal sympathetic nerves in diabetic rats. Effect of i.a. bolus of saline (10 µL), and the serotonergic 5-HT_1/5/7_ receptor type agonists, 5-CT (0.1, 0.4 and 1.0 µg/kg), on the vasopressor responses elicited by electrical stimulation of renal sympathetic nerves in diabetic rats. * *p* < 0.05 vs. saline.

The i.a. administration of 0.4 µg/kg of α-methyl-5-HT (α-m-5-HT), 1-phenylbiguanide (1-PBG), cisapride and AS-19 (5-HT_2_, 5-HT_3_, 5-HT_4_ and 5-HT_7_ receptor agonists, respectively) did not evoke any effect in the electrically induced ΔRPP (Figure 1). The only drug that reproduced serotonin inhibitory action was 5-CT, a 5-HT_1/5/7_ receptor agonist (Figure 1). The 5-CT inhibitory effect (0.1, 0.4 and 1.0 µg/kg) was produced in a dose- and frequency-dependent way (Figure 2), as occurred with 5-HT.

### 2.4. Changes on the Electrically Induced ΔRPP by the i.a. Administration of 5-HT_1_ Receptor Subtype Agonists in Diabetic Rats

**Figure 3:**
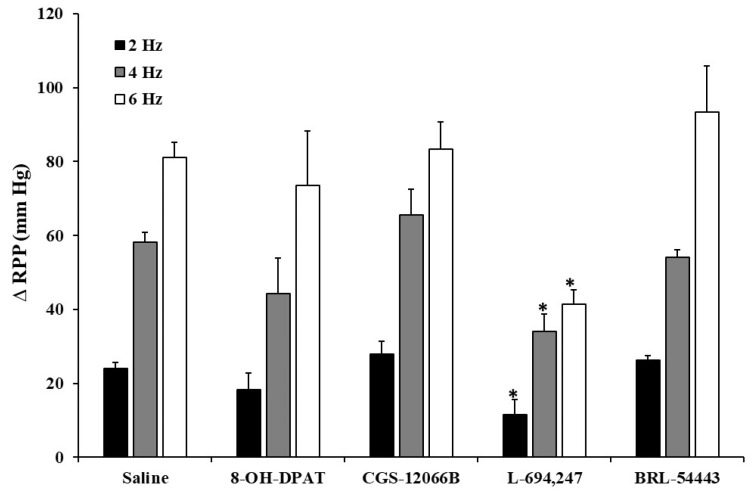
Effect of i.a. bolus administration of 5-HT_1_ receptor subtype agonists on the renal vasopressor responses in diabetic rats. Effect of i.a. bolus of saline (10 µL), and the serotonergic receptor subtype agonists 8-OH-DPAT (5-HT_1A_), CGS-12066B (5-HT_1B_), L-694,247 (5-HT_1D_), and BRL-54443 (5-HT_1F_) (0.4 µg/kg each agonist), on the vasopressor responses elicited by electrical stimulation of renal sympathetic nerves in diabetic rats. * *p* < 0.05 vs. saline.

Intraarterial administration of neither 8-OH-DPAT, CGS-12066B, nor BRL-54443 (5-HT_1A_, 5-HT_1B_ and 5-HT_1F_, respectively, at a dose of 0.4 µg/kg each) induced any change in the electrically induced renal vasopressor responses in diabetic rats (Figure 3). In contrast, only the i.a. bolus administration of L-694,247 (0.4 µg/kg), a selective 5-HT_1D_ receptor agonist, was able to reproduce the 5-HT and 5-CT inhibition of the ΔRPP produced by renal periarterial nerve stimulation at all stimulation frequencies (Figure 3).

### 2.5. Influence of Intravenous Administration of SB 699551 or LY310762 on the Saline or 5-CT Effect on the Renal Vasopressor Responses Induced by Electrical Stimulation in Diabetic Rats

The i.v. administration of neither the selective 5-HT_5A_ receptor antagonist (SB 699551) nor the selective 5-HT_1D_ antagonist (LY310762) (1 mg/kg each one) (nor their vehicle, saline 1 mL/kg i.v.) modifies per se the renal pressor responses induced by sympathetic electrical stimulation in diabetic rats.

**Figure 4:**
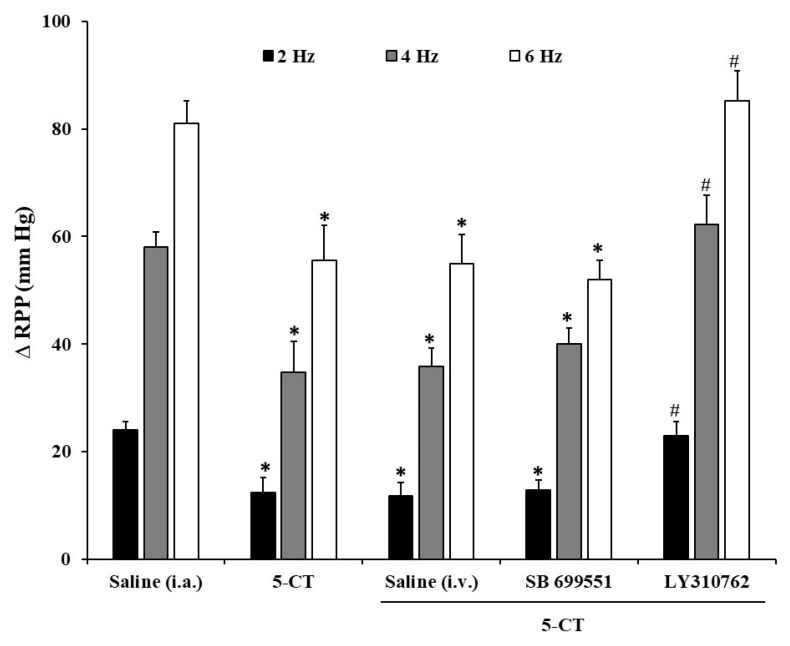
Effect of i.v. pretreatment of serotonergic receptor subtype antagonists on the 5-CT inhibitory effect of the renal sympathetic-induced vasopressor responses in diabetic rats. Effect of i.a. bolus of saline (10 µL) or 5-CT (0.4 μg/kg) in the absence or the presence of i.v. pretreatment with saline (1 mL/kg), SB 699551, or LY310762 (1 mg/kg each) on the vasopressor responses elicited by electrical stimulation of renal sympathetic nerves in diabetic rats. * *p* < 0.05 vs. saline i.a.; ^#^ *p* < 0.05 vs. 5-CT.

In any case, i.a. administration of 0.4 μg/kg of 5-CT in the presence of SB 699551 induced an inhibition of the vasopressor response (Figure 4), which means that 5-HT_5A_ is devoid of any action on the inhibitory effect produced by 5-CT.

The i.v. administration of the 5-HT_1D_ antagonist, LY310762, was able to block the inhibitory effect provoked by 5-CT on the sympathetic vasopressor responses in the kidney of diabetic rats (Figure 4).

### 2.6. Role of Saline or L-694,247 (5-HT_1D_ Receptor Agonist) on the Renal Vasopressor Responses Induced by i.a. Administration of Exogenous Noradrenaline (NA) in Diabetic Rats

**Figure 5:**
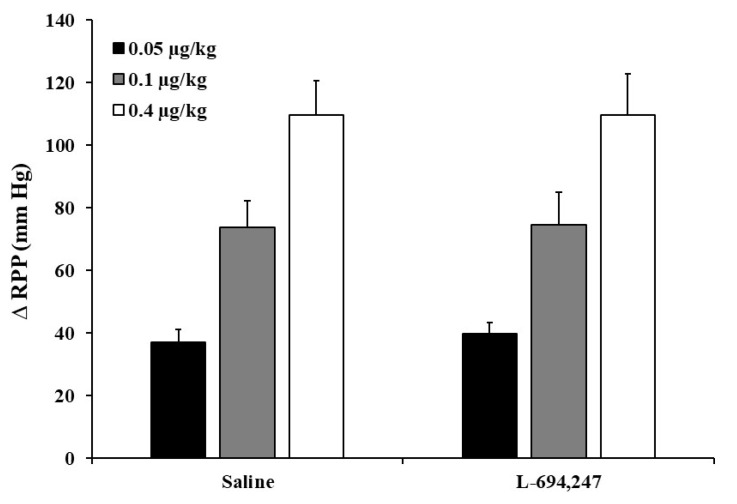
Effect of L-694,247 on the noradrenaline-induced increases in renal perfusion pressure in diabetic rats. Effect of i.a. bolus injections of saline (10 µL) or L-694,247 (0.4 µg/kg) on the increases in renal perfusion pressure (ΔRPP) evoked by i.a. administration of exogenous noradrenaline (0.05, 0.1 and 0.4 µg/kg) in the in situ-autoperfused kidney of diabetic rats. No statistical significance.

The dose-response (D-R) curve E’0 induced by the i.a. administration of NA was 37.1 ± 3.7, 73.8 ± 10.3, and 109.6 ± 13.2 mm Hg at NA i.a. bolus of 0.05, 0.1, and 0.4 µg/kg doses, respectively. The increase in RPP (D-R curve E’0) caused by the i.a. administration of exogenous NA (control group) remained stable after i.a. bolus of saline (Figure 5). Interestingly, i.a. bolus of L-694,247 (0.4 μg/kg) failed to inhibit the pressor responses to i.a. administration of exogenous NA (Figure 5).

### 2.7. Influence of Intravenous Administration of Vehicle, ODQ, Glibenclamide, or Indomethacin on the Saline- or L-694,247-Effect on the Renal Vasopressor Responses Induced by Electrical Stimulation in Diabetic Rats

The i.v. bolus of neither the vehicles (saline, PEN; 1 mL/kg each), indomethacin (2 mg/kg), glibenclamide (20 mg/kg), nor ODQ (10 μg/kg) alters per se the ΔRPP induced by electrical stimulation at 2, 4, and 6 Hz.

**Figure 6:**
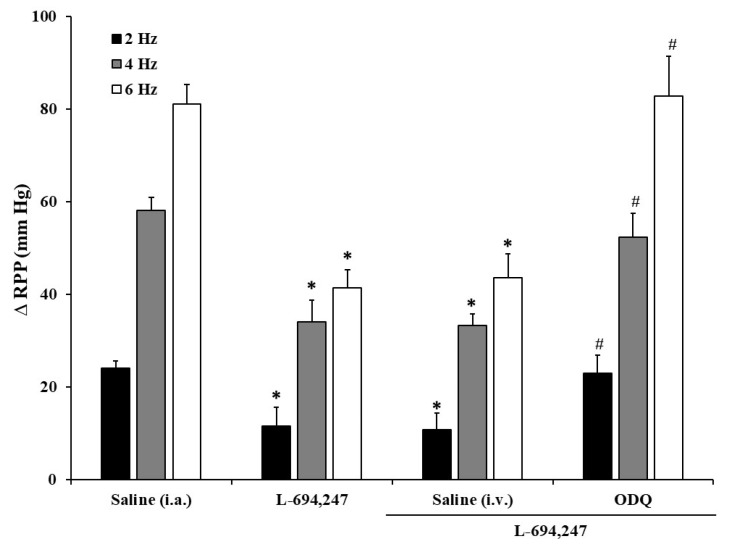
Effect of i.v. administration of ODQ on the 5-HT_1D_ agonist inhibitory action of the renal vasopressor responses in diabetic rats. Effect of i.a. bolus of saline (10 µL) or L-694,247 (0.4 μg/kg) in the absence or the presence of i.v. pretreatment with saline (1 mL/kg) or ODQ (10 μg/kg) on the vasopressor responses elicited by electrical stimulation of renal sympathetic nerves in diabetic rats. * *p* < 0.05 vs. saline i.a.; ^#^ *p* < 0.05 vs. L-694,247.

L-694,247 inhibition (0.4 μg/kg, i.a.) of electrically induced renal vasoconstrictions was exclusively abolished by i.v. administration of 10 µg/kg of the guanylyl cyclase inhibitor, ODQ (Figure 6). In contrast, both the i.v. administration of glibenclamide (a specific inhibitor of ATP-sensitive K^+^ channels) and indomethacin (a non-selective cyclooxygenase (COX) inhibitor) failed to reverse the L-694,247 inhibitory action on the renal vasopressor responses of diabetic rats.

## 3. Discussion

**Figure 7:**
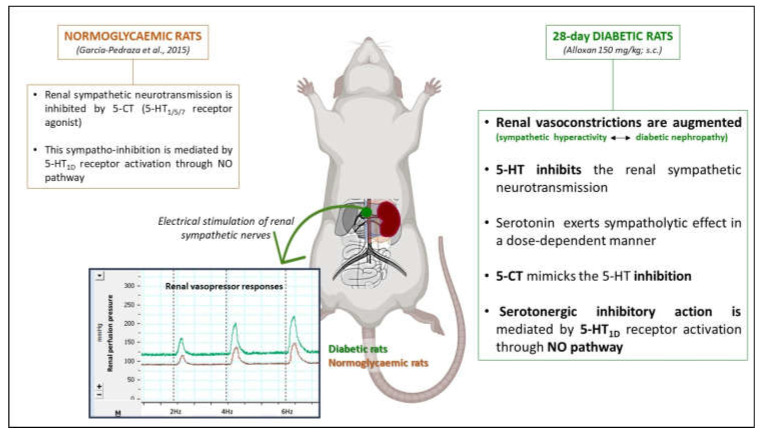
Scheme summarizing the main outcomes in 5-HT modulation of the renal sympathetic neurotransmission in normoglycaemic and diabetic animals [18].

Our work brings to light that diabetes in rats augments renal vasopressor responses induced by sympathetic input, exposing that serotonergic inhibition of noradrenergic outflow, in the in situ-autoperfused rat kidney, is mediated by 5-HT_1D_ receptor subtype activation that involves the NO pathway (Figure 7).

To study these actions, we used a chemically induced diabetic model in rats [27]. Alloxan administration induces a pattern similar to human type 1 diabetes due to its selective damage to the pancreatic β-cells in the islets of Langerhans that produce insulin. This drug caused increased glycaemia after day 2 post-injection, with a consequent loss of weight. Hyperglycaemia stayed at the same rate during the 28-day period, whereas weight slightly increased, reaching values similar to those prior to alloxan administration [17,25,26].

After anaesthesia, our technique of in situ autoperfusion of rat kidney is a specific in vivo experimental model, first tuned up by Fink and Brody (1978) [28] and modified by us [16,18], which continuously measures SBP, HR, and renal blood flow, assessing quick changes in the latter due to i.a. drug administration or to sympathetic periarterial renal nerve stimulation. Basal SBP in diabetic animals (current data) is similar to normoglycaemic animals [18]; however, RPP is significantly higher, while HR is diminished. These differences may be explained by the renal damage originated by the hyperglycaemia, which is usually accompanied by renal microvascular alterations, glycosuria [29], and heart compensatory mechanisms that lead to alterations in HR. Although the development of hypertension during diabetes is well-known, in our 28-day diabetic model, in anaesthetized rats, there is no change in SBP compared to normoglycaemic animals [18], probably due to the duration of diabetic state, since we have already described increased SBP in a 56-day diabetic model using the same experimental technique [30]. None of the i.v. or i.a. administered drugs modified these basal parameters, with the exception of 5-HT at high doses and α-m-5-HT (5-HT_2_ receptor agonist), which augmented RPP, coming back to basal values within 2 min. This fact has been previously shown by us [18] and may be explained by the vasoconstrictor role of this 5-HT receptor type at renal level [16,30,31,32].

The renal vascular bed is a master modulator of blood pressure homeostasis. Thus, diabetic nephropathy originates renal dysfunction that may induce increased renal vascular resistance and, subsequently, chronic kidney damage, that runs in hypertension and vascular altered homeostasis. In this sense, the adrenergic system plays a crucial role, since sympathetic nerves extensively innervate the kidney [33,34]. Renal nerves follow the renal arteries and innervate not only the vasculature but also the juxtaglomerular apparatus and the basement membrane of epithelial cells within the nephron. Thus, electrical stimulation of renal periarterial nerves induces frequency-dependent increases in RPP due to NA release [18]. The ΔRPP are augmented in diabetic animals in comparison with normoglycaemic individuals [18], which suggests the development of diabetic neuropathy, with a marked sympathetic hyperactivity that, in the long term, exacerbates cardiovascular risk in diabetic patients [35,36]. In addition, the fact that increases in renal vasopressor responses after i.a. exogenous NA are higher in diabetic than in normoglycaemic animals supports both the development of diabetic peripheral neuropathy and microvascular complications in the kidney [37,38]. Our group has already shown cardiovascular sympathetic overactivity as a marker of cardiovascular impairment in diabetes [22,25,26] or hypertension [39].

The serotonergic system acts as modulator, both potentiating and inhibiting noradrenergic or vagal outflow at cardiac and vascular levels in different experimental models [21,22,23,24,40,41]. Furthermore, our group has described the serotonergic inhibitory action of noradrenergic input at mesenteric and renal levels [18,19,39]. Moreover, in diabetic animals, 5-HT is also able to reduce vasopressor responses to sympathetic stimulation in the kidney (current data). In any case, it is important to remark that serotonin doses utilized are higher than in normoglycaemic animals [18], probably due to diabetic damage both at neurohumoral and microvascular levels [2,3,4], which would make it necessary to increase serotonin doses to be able to reduce vasopressor responses induced by sympathetic hyperactivity [8,9]. These results show the importance of the peripheral effects of this biogenic amine, and in general, of the whole serotonergic system, and open new frontiers in the search for novel treatments in pathologies, such as renovascular and neuropathic diabetic complications.

To further analyse the 5-HT receptor type involved in this serotonergic inhibitory effect on renal noradrenergic input at vascular level, we administered selective agonists (i.a. bolus). Our results allowed us to conclude that only the selective 5-HT_1/5/7_, 5-CT, was able to reproduce the serotonin inhibitory effect in a dose-dependent manner as serotonin did, as occurred in the normoglycaemic model [18], whereas neither α-methyl-5-HT, 1-PBG, cisapride, nor AS-19 (5-HT_2_, 5-HT_3_, 5-HT_4_ and 5-HT_7_ receptor agonists, respectively) modified the electrically induced renal vasopressor responses. These data differ from the serotonergic sympatholytic effect at vascular level in a pharmacological-altered model, where both 5-HT_1_ and 5-HT_7_ receptor types were involved [24] in the inhibitory action. As currently, no selective 5-HT_5A_ receptor agonist is described, to study this receptor type effect, we utilised a 5-HT_5A_ selective serotonergic antagonist (SB 699551) prior to 5-CT administration [25]. As SB 699551 did not reverse 5-CT inhibitory action, we could devoid 5-HT_5A_ receptor type of any sympatholytic effect in the kidney vasculature of diabetic animals and confirm that the sole serotonergic receptor type involved in the sympatho-inhibitory effect on vasopressor responses is the 5-HT_1_ receptor type, which is in agreement with previous results by our group at vascular level in normoglycaemic and diabetic rats [21,22], and at renal or mesenteric levels in normoglycaemic rats [18,19], but differs from those obtained in the mesentery of hypertensive rats, where 5-HT_4_ was the responsible receptor type of the serotonergic inhibitory effect [39].

In the search for more specific therapeutic approaches in diabetic complications, we determined the 5-HT_1_ receptor subtype/s involved in this inhibitory effect, and established that only the receptor 5-HT_1D_ is implicated in serotonergic inhibitory effect on electrically induced renal vasoconstrictions, since L-694,247 (5-HT_1D_ agonist) [42] reproduced 5-HT and 5-CT inhibitory action, but this selective 5-HT_1D_ agonist was not able to reduce vasoconstrictor responses induced by i.a. administration of exogenous NA; thus, we propose a prejunctional locus for 5-HT_1D_ receptors. To confirm these results, we i.v. administered LY310762 (a 5-HT_1D_ receptor antagonist) [43] prior to i.a. bolus of 5-CT, which completely blocked its sympatholytic effect. Hence, we disclose that prejunctional 5-HT_1D_ receptor activation is the main factor responsible for the serotonergic sympatholytic effect at renal level in diabetic rats. The 5-HT_1D_ receptor, at prejunctional locus, has already been described as sympatholytic at the renal level [18], and at cardiac and vascular levels, in pithed rats as well [21,40] in normoglycaemic animals. In any case, it is worthwhile to mention that short-term diabetic state modifies the serotonergic profile involved in the sympatho-modulation, unmasking peripheral prejunctional 5-HT_1A_ and 5-HT_5A_ at vascular and cardiac level, respectively, in pithed rats [22,41].

Endothelial dysfunction contributes to the pathogenesis of microvascular diabetic complications. Thus, considering that (a) endothelial relaxation depends on several agents such as NO [44], prostacyclin [45], and/or endothelium-derived hyperpolarizing factor (EDHF) [46], (b) endothelial-dependent vasorelaxation is altered in diabetes [47], and (c) the NO synthesis was involved in 5-HT_1D_ renal sympatho-inhibitory effect in normoglycaemic rats [18], we determined the possible role of these vasodilator agents in the serotonergic inhibitory action in diabetic rats using ODQ (a guanylyl cyclase inhibitor), indomethacin (a COX_1/2_ inhibitor), or glibenclamide (an ATP-sensitive K^+^ channels blocker) [48,49,50]. As was observed in normoglycaemic animals, only the NO pathway is involved in the 5-HT_1D_ renal sympatholytic action in diabetes, which is also in agreement with previous results by us in the diabetic pithed rat model [22,51]. Although there is evidence that, during diabetes, microvascular endothelial dysfunction is primarily characterized by impaired endothelial repair due to enhanced oxidative stress and a decreased release of NO [52], our present results show that at renal level, the NO pathway maintains its relevant role in mediating serotonergic modulation of noradrenergic-induced vasopressor responses.

This work has some constraints: taking into account our experience, experiments were only performed in males; thus, there is no sex bias. Although haemodynamic parameters (SBP and HR) are in the normal range for anaesthetized rats, our technique requires invasive surgery, and finally, we should mention that renal sympathetic nerve activity is not directly measured, but indirectly estimated through the induced changes in renal perfusion pressure. In any case, it is noteworthy that this work, in basic pharmacology, has highlighted that serotonergic modulation of NA release at renal level, via 5-HT_1D_ receptor activation and, consequently, NO pathway in diabetic individuals, may be a therapeutic approach to reduce sympathetic hyperactivity closely tied to renal microvascular complications. Currently, renal sympathetic denervation has become, despite its side effects (renal artery injury and deterioration of kidney function, among others), one of the treatment options in pathologies with increased sympathetic nerve activity, such as cardiovascular complications of diabetes or hypertension [53,54]. Our results set down the basis to use serotonergic inhibition of noradrenergic outflow as a feasible alternative to complete renal sympathetic ablation in cardiovascular and renal complications derived from diabetes.

## 4. Materials and Methods

### 4.1. Drugs Employed

The compounds and suppliers used in the experiments described throughout the manuscript were: sodium pentobarbital (Dolethal^®^; Vetoquinol, Madrid, Spain); heparin sodium from Rovi (Madrid, Spain); atropine sulphate (Scharlau, Barcelona, Spain); indomethacin (Acofarma, Barcelona, Spain); alloxan monohydrate, 1-PBG, BRL-54443 maleate salt, glibenclamide, NA bitartrate, and 5-HT hydrochloride (Merck Life Sciences S.L.U., Madrid, Spain); 5-CT, 8-OH-DPAT, α-methyl-5-HT, AS-19, cisapride, CGS-12066B, L-694,247, LY310762 hydrochloride, ODQ, and SB 699551 (Tocris Bioscience, Bristol, UK).

All the compounds were dissolved in saline at the time of experimentation, except AS-19 (dissolved in EtOH 5%), cisapride (0.01 M HCl), and both indomethacin and glibenclamide (dissolved in a mixture of PEN). None of the vehicles alter either RPP or SBP.

### 4.2. General Methods

A total of 135 male Wistar rats (275–325 g) became diabetic by a single subcutaneous injection of alloxan (150 mg/kg) and were housed for 28 days in a 12/12 h light–dark cycle under specific temperature (22 ± 2 °C) and humidity (50%) conditions, with free availability of food and tap water.

Before alloxan administration and until day 28, animals were weighed and non-fasting blood glucose levels were periodically measured with a glucometer (Accu-chek^®^ Aviva, Roche Diagnostics; Barcelona, Spain). Rats with blood glucose levels <250 mg/dL (non-diabetic) were discarded.

After 28 days of the diabetes induction, animals were anaesthetized (sodium pentobarbital, 60 mg/kg, i.p.) and prepared for the in situ autoperfusion of left kidney as previously described by our group [16,18]: catheters were placed in the trachea, the right and left carotid arteries (to measure SBP and HR, and RPP, respectively), and jugular and femoral veins (for i.v. administration).

The in situ perfusion of the left kidney was performed according to the method of Fink and Brody (1978) [28] modified by Morán et al. (1997) [31]. The renal vascular bed was perfused using an extracorporeal circuit and a constant flow Gilson peristaltic pump from the left carotid artery to the left renal artery [16,18,28,30,55], which was exposed by midline laparotomy and deflection of the intestines to the right side of the animal. A loose tie was placed around the aorta 1 cm below the left renal artery and 1 cm above the iliac bifurcation. To prevent blood clots, heparin sodium was administered (5 mg/kg, i.v.) and intravenous infusion of saline (0.9% NaCl) was initiated at a rate of 2 mL/h and continued thought the experiment. Atropine (1 mg/kg) was administered intravenously before the saline infusion in order to block potential cholinergic effects.

Both the distal portion of the external circuit coming from the left carotid and the right carotid were connected to two different pressure transducers connected to an e-corder 410 amplifier (Model ED410, Cibertec, Spain) for measurement of the RPP and SBP and HR, respectively.

At the beginning of each experiment, the flow was adjusted to make the RPP equal to the SBP. Flow was constant throughout the experiments; thus, variations in the RPP reflected variations in renal vascular resistance. The flow rate through the renal vascular beds ranged from 2 to 2.9 mL/min [18].

**Figure 8:**
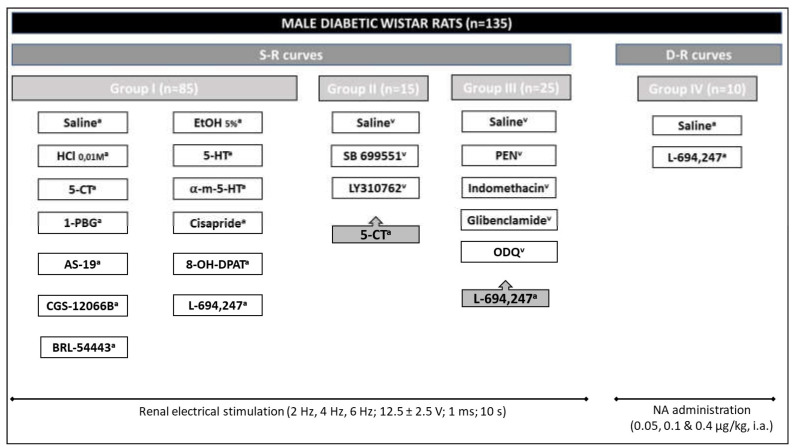
Scheme of experimental protocols, showing the number of animals used. Experimental protocols showing the two main sets of diabetic animals and the different groups used in these experiments, in which renal vasopressor responses are obtained by renal nerve sympathetic stimulation (S-R curves) or i.a. bolus of noradrenaline (NA) (D-R curves). ^a^ i.a. administration; ^v^ i.v. administration; α-m-5-HT: α-methyl-5-HT; S-R: (electrical) stimulus-response; D-R: dose-response (to exogenous i.a. NA); PEN: Polyethylene glycol/Ethanol/NaOH 33:33:34.

The diabetic animals were initially divided into two main clusters (see Figure 8), so that the effects produced by different 5-HT agents could be investigated on the vasopressor responses induced by: (i) electrical stimulation of sympathetic renal nerves (S-R group, n = 125); or (ii) i.a. bolus injections of exogenous NA (D-R group, n = 10). In the vasopressor S-R curves and D-R curves elicited by electrical stimulation and exogenous NA, respectively (see Figure 8), each response was elicited under unaltered values of resting blood pressure. The electrical stimuli (12.5 ± 2.5 V; 1 ms; 2, 4 and 6 Hz), as well as dosing with NA (0.05, 0.1 and 0.4 µg/kg), were given using sequential schedule at 3–5 min intervals. At each frequency, stimulation was continued until the response was maximal (5–10 s), and basal perfusion pressure was restored immediately after interruption of the stimulation.

### 4.3. Experimental Protocols

After the animals had been in a stable haemodynamic condition for at least 15 min, baseline values of SBP, HR, and RPP were measured.

#### 4.3.1. Electrical Stimulation of the Periarterial (Vasopressor) Renal Nerves

The first main group (S-R curves; Figure 8) was designed to study the influence of 5-HT agonists and antagonists on renal sympathetic neurotransmission in diabetic rats. In addition, the implication of possible indirect pathways on 5-HT effect was determined.

Increases in RPP were obtained by the electrical stimulation of the periarterial renal nerves, by placing a small bipolar electrode close to the origin of the left renal artery connected to a Cibertec Stimulator CS-9 using square wave pulses at increasing stimulation frequencies (2, 4 and 6 Hz). In that way, the control S-R curve (E0) was completed in 15 min. Afterwards, the rats were divided into three different groups. The first one (Group I; n = 85; Figure 8) received intraarterial bolus injections of a maximum volume of 10 µL using a micro-syringe via the distal cannula: (a) saline, (b) ethanol 5%, (c) HCl 0.01 M, (d) 5-HT (0.0125, 0.1 and 0.4 µg/kg), (e) 5-CT (5-HT_1/5/7_ agonist; 0.1, 0.4 and 1.0 µg/kg) and the following agonists at a dose of 0.4 µg/kg: α-methyl-5-HT (5-HT_2_), 1-PBG (5-HT_3_), cisapride (5-HT_4_), AS-19 (5-HT_7_), 8-OH-DPAT (5-HT_1A_), CGS-12066B (5-HT_1B_), L-694,247 (5-HT_1D_), and BRL-54443, (5-HT_1F_). After 5 min of the corresponding i.a. administration, a new S-R curve (E1) was obtained as described above for the S-R curve E0.

The second cluster (Group II; n = 15; Figure 8) was carried out to confirm the 5-HT receptors involved in the serotonergic modulation of the renal sympathetic nerve activity. These animals were administered i.v. vehicle (saline, 1 mL/kg) or 1 mg/kg of the following antagonists, SB 699551 (5-HT_5A_) or LY310762 (5-HT_1D_). The corresponding curves (E0_saline_, E0_SB 699551_ and E0_LY310762_) were completed after 10 min, respectively. Then, the animals received an i.a. administration of 5-CT (0.4 µg/kg). After 5 min of i.a. administration, a new S-R curve (E1) was obtained.

The third group (Group III; n = 25; Figure 8) was performed to determine the indirect pathways involved in the serotonergic effect on renal sympathetic nerve activity. These animals received, intravenously, as follows: (a) saline (1 mL/kg), (b) PEN (1 mL/kg), (c) a non-selective COX inhibitor, indomethacin (2 mg/kg), (d) an ATP-sensitive K^+^ channels blocker, glibenclamide (20 mg/kg), or (e) a selective inhibitor of soluble guanilyl-cyclase, ODQ (10 µg/kg), 10 min before its corresponding S-R curve. After that, the animals received an i.a. bolus of L-694,247 (0.4 µg/kg) to obtain a new S-R curve (E1).

#### 4.3.2. Administration of Exogenous NA

In another set of animals (Group IV; Figure 8; n = 10) prepared as described above, the bipolar electrode was omitted and D-R curves by intraarterial administration of exogenous NA (0.05, 0.1 and 0.4 µg/kg) were constructed before (E’0) and 5 min after (E’1) administration of i.a. saline (10 µL) or L-694,247 (0.4 µg/kg).

### 4.4. Data Presentation and Statistical Procedures

All the data in the text, tables, and figures, are presented as mean ± SEM of five experiments. Changes in renal vascular resistance are stated as increases in RPP (mm Hg), in comparison with the corresponding baseline value. Statistical significance was carried out with one-way ANOVA followed by Student–Newman–Keul’s post hoc test. Statistical significance was accepted at *p* < 0.05.

## 5. Conclusions

This study discloses that twenty-eight-day experimental diabetes originates an enhancement of the renal sympathetic activity and that the serotonergic system modulates this sympathetic drive: activation of prejunctional 5-HT_1D_, through NO pathway, reduces noradrenergic-evoked vasopressor responses in the kidney. Thus, this study enlarges the knowledge of complex regulation mechanisms occurring in renal diabetic complications (including both neuropathy and microvascular alterations), and unmasks new therapeutic approaches based on 5-HT_1D_ receptor activation to diminish renal sympathetic outflow and, therefore, tackle diabetic renal disturbances.

## Figures and Tables

**Table 1:** Effect of saline and 5-HT on the increases in renal perfusion pressure induced by renal sympathetic stimulation in diabetic rats.

Compounds		Stimulation Frequencies (Hz)		
		2	4	6
**Saline** (10 µL)		*24.0 ± 1.6*	*58.1 ± 2.7*	*81.0 ± 4.2*
**5-HT** (µg/kg, i.a.)	**0.0125**	19.5 ± 4.2 *	40.6 ± 7.9 *	61.0 ± 9.2 *
	**0.1**	16.2 ± 2.8 *	32.7 ± 3.8 *	53.7± 6.3 *
	**0.4**	11.8 ± 6.0 *	26.3 ± 9.7 *	51.2 ± 9.9 *
		***ΔRPP (mm Hg)***		

Effect of i.a. bolus injections of saline (10 µL, n = 5) or 5-HT (0.0125, 0.1 and 0.4 µg/kg; n = 5 for each treatment) on the increases in renal perfusion pressure (ΔRPP) evoked by renal sympathetic stimulation (2, 4 and 6 Hz) in the in situ-autoperfused kidney of diabetic rats. * *p* < 0.05 vs. saline.

## Data Availability

The main data are included in this manuscript. All data are available from the corresponding author on reasonable request.
